# Investigations on the Thermodynamics Characteristics, Thermal and Dielectric Properties of Calcium-Activated Zinc-Containing Metallurgical Residues

**DOI:** 10.3390/ma15030714

**Published:** 2022-01-18

**Authors:** Aiyuan Ma, Xuemei Zheng, Lei Gao, Kangqiang Li, Mamdouh Omran, Guo Chen

**Affiliations:** 1 School of Chemistry and Materials Engineering, Liupanshui Normal University, Liupanshui 553004, China; may_kmust11@163.com (A.M.); zxm_lpssy19@163.com (X.Z.); 2 Guizhou Provincial Key Laboratory of Coal Clean Utilisation, Liupanshui Normal University, Liupanshui 553004, China; 3 Key Laboratory of Green-Chemistry Materials in University of Yunnan Province, Kunming Key Laboratory of Energy Materials Chemistry, Yunnan Minzu University, Kunming 650500, China; glkust2013@hotmail.com; 4 Key Laboratory of Aerospace Materials and Performance, Ministry of Education, School of Materials Science and Engineering, Beihang University, Beijing 100191, China; 5 Process Metallurgy Research Group, Faculty of Technology, University of Oulu, 90570 Oulu, Finland; 6 Central Metallurgical Research and Development Institute (CMRDI), Cairo 11421, Egypt

**Keywords:** zinc-containing residues, calcium activation, thermal conductivity, dielectric properties, microwave heating

## Abstract

An activate pretreatment of zinc-containing metallurgical residues were proposed by adding CaO and introducing microwave heating approach into the CaO activation pretreatment process to realize the conversion of refractory ore phases into pre-treated ore phase. Thermodynamic characteristics analysis indicated that adding CaO can realize the conversion of refractory ore phases, with the same effect as the carbon additives. Thermal conductivity properties analysis denoted that the thermal conductivity properties of ZnS and ZnFe_2_O_4_ were relatively poor. Meanwhile, the thermal conductivity properties of the residues sample added with 25% CaO were significantly superior to the residues added with other CaO contents, with the maximum specific heat value of 1.348 J/g·K at 350 °C. Dielectric properties analysis highlighted that adding CaO with the dielectric constant properties significantly higher than that of other substances can enhance the microwave absorption capacity of zinc-containing residues. The decrease in dielectric loss and loss tangent value with the increase of temperature and the residues having large microwave penetration depth guaranteed to obtain better uniformity of microwave heating. Furthermore, adding 25% CaO promoted the microwave penetration depth of the residues sample increased in the range of 300–500 °C. This work can lay a theoretical research foundation for solving the key difficulty for efficient Zn recovery from complex zinc-containing metallurgical residues.

## 1. Introduction

Zinc (Zn) endows superior calenderability, corrosion resistance, and wear resistance; thereby it is widely applied in the fields like non-ferrous metallurgy, batteries, automobiles, and building materials [1,2,3]. Sphalerite (ZnS) is the main mineral used in industrial preparation of zinc, via an oxidization roasting approach. With the prosperous development of society, the demand for zinc-based materials has increased year by year, which has exacerbated the increase in the mining volume of sphalerite and the decline in grade [4]. Therefore, considering the increasing mining volume of zinc ore resources and the importance of processing other zinc-containing hazardous waste resources environmental-friendly, developing and utilizing other zinc-containing hazardous waste resources such as metallurgical residues has significant and sound research value. The residues are produced from the smelting process of metals such as zinc (Zn), lead (Pb), iron (Fe), and copper (Cu), and its output is huge and rising sharply [5,6]. Meanwhile, it is a fact that the content of zinc and other elements in the residues exceeding the limit value will damage the furnace cavity’s refractory materials and shorten the furnace’s service life, further to render the decrease in furnace’s productivity and difficult operation [7,8]. Furthermore, residues are classified as hazardous materials, rich in heavy metals (including Zn, Fe, and Cu) and toxic components (including Pb, Cr, Cd, As, and Hg) [9,10]; thereby simple burial and inventory disposal are inadvisable for residues. Therefore, there is an urgent need to develop a clean, efficient and economical method for recovering Zn from metallurgical residues.

The residues contain various conventional smelting metals and precious metals, having high recycling value [11,12]. However, attributed to the diverse compositions and complex structures of the residues, various metal oxides, chlorides, carbon-containing compounds and gangues are doped and wrapped together [13,14], making the recovery process difficult [15,16]. The hydrometallurgy leaching method is more suitable for recycling residues with diverse compositions and complex structures than pyrometallurgy method [17]. Whereas, the application of conventional acid extraction approach is limited by the disadvantages of high acid consumption, high energy consumption, long purification time, low electrolytic zinc quality, and low recovery rate [18,19,20,21]. Hence, the current research hotspots about Zn extraction from zinc-containing resources focus on applying alkaline leaching systems to extract zinc from single-phase zinc-containing minerals, such as zinc oxide ore, iron-zinc ore, and hydrozincite. The commonly reported ammonium salts include ammonium chloride, ammonium sulfate, and ammonium bicarbonate [5,22,23]. The essence of alkaline leaching method is that ammonia compounds can form [Zn(NH_3_)_4_]^2+^ coordination compounds with zinc metal ions (Zn^2+^) to prevent impurities containing Fe, Al and Si from entering the leaching solution, further achieving the effective separation of zinc and impurities [24,25]. Rao et al. [26] found that adding nitrosotriacetic acid (N(CH_2_COOH)_3_, i.e., NTA) into NH_4_Cl-NH_3_ solution can effectively enhance the Zn extraction process. The addition of carboxylate anion (RCOO-) promotes [Zn(NH_3_)_4_]^2+^ coordination compounds to be transformed into more stable [Zn(NTA)(NH_3_)_2_]^−^ coordination compounds, thereby effectively improving the zinc leaching rate.

The previous work has confirmed that adding CH_3_COONH_4_ into NH_3_-H_2_O system is efficient for promoting the zinc extraction from zinc-containing residues [26]; however, zinc ferrite (ZnFe_2_O_4_), zinc silicate (Zn_2_SiO_4_), and zinc sulfide (ZnS) are difficult to leach under the NH_3_-CH_3_COONH_4_-H_2_O system, which causes the Zn leaching rate was low with only 83.76% [24,27]. On the other hand, the successful applications of microwave heating instead of conventional heating to open inclusions ore and complex ore pretreatment process have been reported continuously [28,29,30,31,32]. Therefore, this study proposed an activate pretreatment of zinc-containing residues by adding low-cost and widely-sourced calcium oxide (CaO), and intended to introduce microwave heating technology into the CaO activation pretreatment process of zinc-containing residues to realize the conversion of refractory ore phase (including ZnS, ZnFe_2_O_4_, and Zn_2_SiO_4_) into pre-treated ore phase, further to improve Zn extraction rate. In detail, the calcification thermodynamics characteristics of the residues were explored. The changes of the thermal diffusion coefficient, thermal conductivity, and specific heat of these typical materials (including ZnO, ZnS, ZnFe_2_O_4_, Fe_3_O_4_, KCl, and CaO) in residues and the residues added with different CaO contents (including 5%, 10%, 15%, 20%, 25%, and 30%) at different temperatures were measured; additionally, the dielectric properties of these typical materials and the residues added with different CaO contents were investigated. The microwave heating characteristics of metallurgical residues were studied to helpfully analyze the dielectric properties of the residues.

## 2. Materials and Methods

### 2.1. Materials

The raw materials of metallurgical residues studied in this work were provided by a comprehensive recovery enterprise for secondary zinc resources (Yunnan province, China). The metallurgical residues were a mixture of multiple types of residues, and the residues was dried at 85 °C in an electric heating constant temperature blast drying oven (DHG-9055A, Shanghai Yiheng Scientific Instrument Co., Ltd., Shanghai, China) to a constant weight and then analyzed for chemical composition. The analytical results were shown in Table 1. As determined in Table 1, the residues have complex compositions, with high contents of Zn, Fe, Ca, Cl, and alkaline gangue composition, as well as rare and associated metal In. Hence, the residues raw material exhibits a high recycling value.

**Figure 1:**
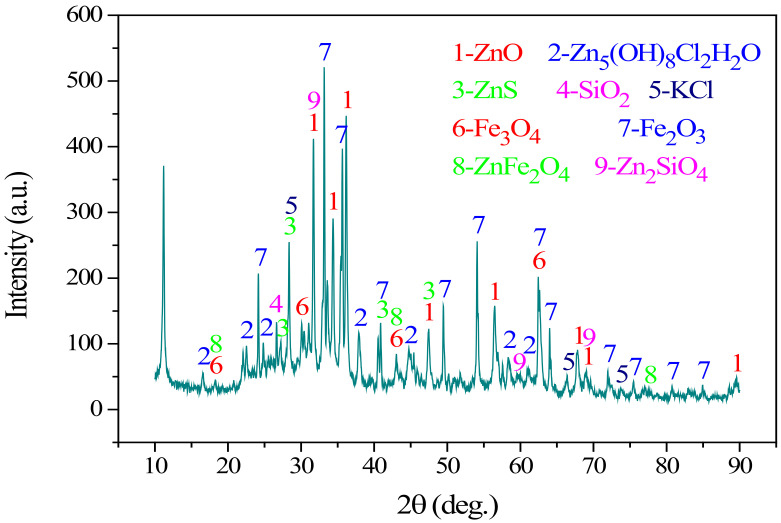
XRD pattern of the residues.

Determining the existing forms of metals ions and impurities in the residues is crucial for the process choice of Zn extraction. The residues were characterized by a rotating target multi-functional X-ray diffractometer (TTRA Ⅲ, Rigaku, Tokyo, Japan). The operation power of the X-ray generator was 18 kW, CuKα irradiation (λ = 1.54056 Å) was applied, the pressure and the current were 40 kV and 200 mA, respectively. Under filtering by a graphite monochromator with high reflection efficiency, scanning was conducted with a scanning rate of 4°/min from 10–90°. The XRD pattern of the residues was illustrated in Figure 1. The XRD pattern revealed that Zn presented as ZnO, Zn_5_(OH)_8_Cl_2_·H_2_O, ZnS, ZnFe_2_O_4_, and Zn_2_SiO_4_, while Fe mainly presented as Fe_3_O_4_ and Fe_2_O_3_. The diversity of the zinc phase and the presence of iron suggest that extracting zinc from the metallurgical residues was very difficult. The activator used in this work, calcium oxide (CaO, analytically pure), was purchased from Tianjin Regent Chemicals Co., LTD (Tianjin, China), with a particle size regime of 60–180 mesh. And before the measurement for the thermal and dielectric properties of calcium-activated zinc-containing metallurgical residues, the mixture samples were prepared by mixing metallurgical residues and calcium oxide with different CaO contents (including 5%, 10%, 15%, 20%, 25%, and 30%) for 10 min in a porcelain mortar.

### 2.2. Measurement Method and Instrument for Thermal Conductivity

**Figure 2:**
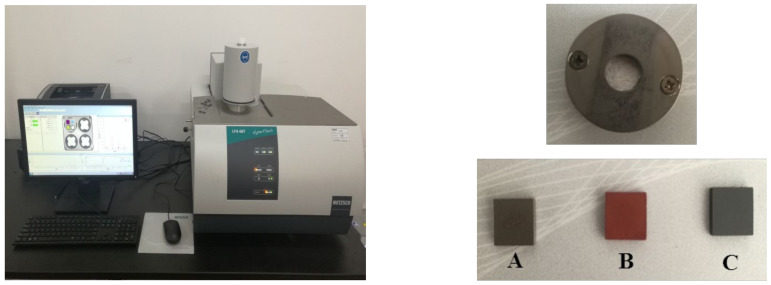
Thermal conductivity measurement system diagram. A—reference sample, B—measured sample, C—graphite-coated sample.

The thermal conductivity performance of minerals is an important factor affecting the heat treatment process, and it is related to those factors such as thermal conductivity (λ), thermal diffusion coefficient (a), and specific heat (c) of the material. In this work, the thermal conductivity parameters of the metallurgical residues were measured using the laser flash analyzer (LFA 467, NETZSCH, Selb, Germany) through the laser flash method (GB/T 22588-2008) [33,34]. The more detailed information about the measurement principle of the laser flash method is provided in Supplementary Materials. The measurement instrument and mold are shown in Figure 2. In the measurement process, the material that is easy to be tableted and formed is firstly tableted, and a 10 × 10 (h) square block is prepared (as the sample B shown in Figure 2). Besides, sample A is a reference sample with similar geometric dimensions, similar thermal properties and a known calorific value. To ensure that the surface light energy absorption ratio and infrared emissivity are the same between the two samples, surfaces of samples A and the sample to be tested (sample B) are coated with graphite at the same time to obtain the sample C. The thermal conductivity parameters of samples are measured under same radiation conditions. Furthermore, it is difficult for ZnO to be compressed into tablets to measure the thermal diffusivity of the powder sample. Therefore, the mold carrying the sample is cylindrical (as shown in the upper right figure in Figure 2), with a diameter of 11 mm and a height of 2 mm. In the case of unsteady heat conduction, there is a problem that the flash point at a specific temperature (T) is difficult to measure during the measurement of the thermal conductivity parameters of zinc silicate (Zn_2_SiO_4_), ferric oxide (Fe_2_O_3_) and the residues added with 30% CaO; hence, the corresponding thermal conductivity parameters are not listed below.

### 2.3. Measurement Method and Instrument for Dielectric Property

**Figure 3:**
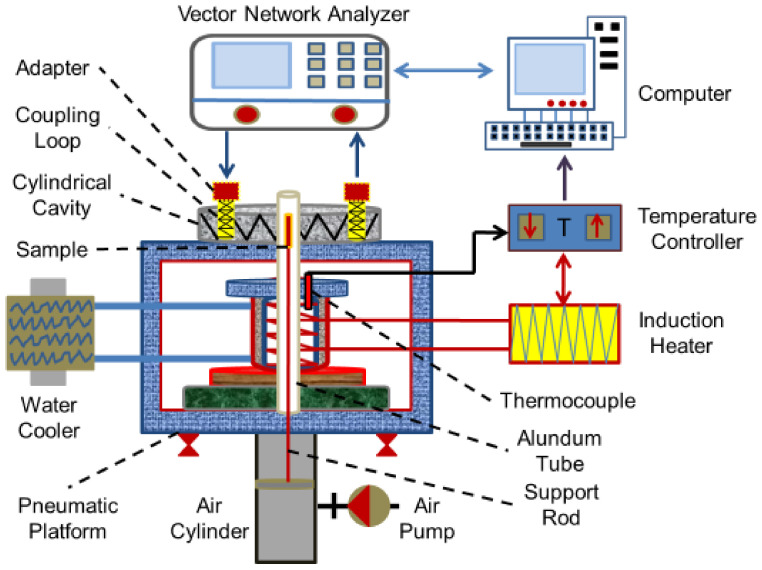
Schematic diagram of high-temperature permittivity measurement system.

The interaction between microwave and the heated material is expressed by complex permittivity [35,36]. The resonant cavity perturbation method is widely used in the measurement of the complex permittivity of solid and liquid materials [37,38]. The more detailed information about the measurement principle of the resonant cavity perturbation method was provided in Supplementary Materials. The schematic diagram of high-temperature permittivity measurement system is displayed in Figure 3. The test system mainly included vector network analyzer, cylindrical cavity, coupling device, waveguide coaxial conversion head, electromagnetic induction heater, quartz sleeve, sample lifting platform, circulating cooling system, and computer software system. The overall technical indicators of the permittivity measurement system were as follows: the service temperature ranged from 0–1000 °C; the microwave frequency is 2450 (±50) MHz; the test value regime covered 1–100 of dielectric constant (ε_r_′) and 5 × 10^−3^ − 1 of loss tangent coefficient (tan δ).

The specific measurement procedure is provided as follows [39,40]:

(1) Calibration of the dielectric parameter test system before testing through air calibration, the dielectric constant of air is 1.00.
(2) Dried residues sample is placed into a flat-bottomed test quartz tube with an inner diameter of 4 mm, an outer diameter of 6 mm, a length of 52 mm, and a wall thickness of 1 mm. The height of the sample in the tube should be kept at about 45 mm.
(3) In the first step, the quality factor will be tested under the cavity condition; in the second step, the quality factor will be tested under the load condition after placing the sample, and the dielectric properties data will be directly output through the calculation program of the system.

Besides, the heating area of the test system and the test chamber are separated to ensure the calibration of the test cavity. The heating area and the test area are connected by a lifting system. The nozzle is plugged with quartz wool to prevent the material from splashing during heating.

## 3. Results

### 3.1. Analysis of Thermodynamic Characteristics

Thermodynamics characteristics analysis can theoretically reveal the influence of CaO additives on the phase transformation of residues, further to determine the feasibility of realizing the conversion of refractory ore phase (including ZnFe_2_O_4_, Zn_2_SiO_4_, and ZnS) to pre-treated ore phase by adding low-cost and widely-sourced calcium oxide (CaO). The pre-treated ore phase is beneficial to improve the Zn extraction rate from residues.

**Figure 4:**
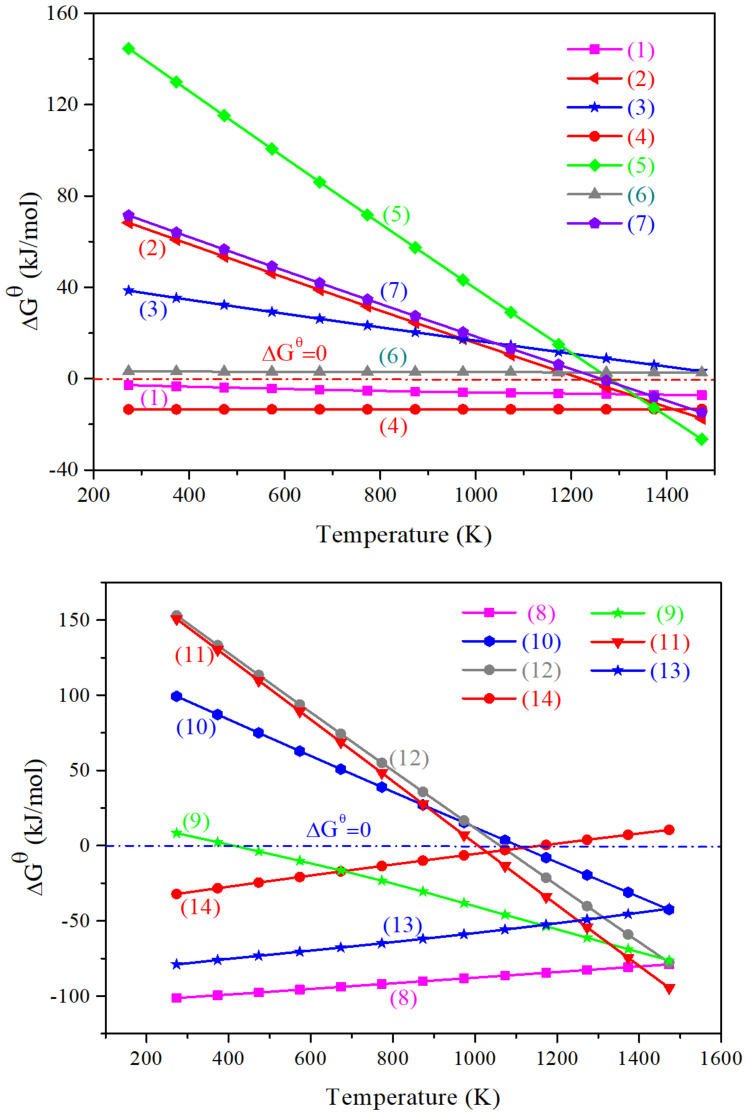
Dependence of ∆G^θ^ for the main reactions (referring to Table 2) in the calcium-activated residues on temperature. The numbers (1)~(14) marked in Figure 4 correspond to the NO. 1~14 in Table 2.

The main reactions probably appeared in the calcium-activated residues is presented in Table 2. The dependence of ∆G^θ^ for the main reactions in the calcium-activated residues on temperature is depicted in Figure 4, wherein the thermodynamic data were calculated by HSC Chemistry thermochemical software. As illustrated in Figure 4, the reaction of ZnS_(s)_ + CaO_(s)_ = ZnO_(s)_ + CaS_(s)_ can hardly react within the temperature range of 0–1200 °C, but it can be seen from Table 2 that ZnS can undergo oxidation reaction (reaction NO. 8) at room temperature. Additionally, it is found from the comparative calculation of the carbothermal reduction reaction conditions that the addition of C reducing agent for the reaction of ZnS_(s)_ + CaO_(s)_ = ZnO_(s)_ + CaS_(s)_ can achieve the conversion of ZnS to ZnO (reaction NO. 7). However, the transition temperature is higher than 1000 °C. Similarly, the reaction transition temperature for Zn_2_SiO_4(s)_ + C_(s)_ is also above 1000 °C (reaction NO. 5); while the reaction transition temperature of ZnFe_2_O_4(s)_ + C_(s)_ is relatively lower than that of ZnS and Zn_2_SiO_4_, with the transition temperature of 550–950 °C (reactions NO. 9–13). Moreover, it is concluded that during the carbothermic reduction reaction of ZnFe_2_O_4_ phase, ZnO can be reduced in a metallic state by C and CO ingredients (reactions NO. 2–3). The volatilization of zinc in gaseous form is not conducive to the centralized recovery of zinc. The reduction reaction generally consumes relatively high energy. In summary, it can be summarized that compared with the carbothermal reduction reaction (with the addition of C), Zn_2_SiO_4_ and ZnFe_2_O_4_ phases can also undergo mineral phase conversion under the roasting conditions with the addition of CaO. Meanwhile, ZnS phase can also undergo oxidation reactions in the air atmosphere during roasting. Therefore, investigating the influence of roasting temperature on the transformation of refractory mineral phase in residues added with CaO is of great significance in energy saving and consumption reduction and the recovery of valuable metal zinc.

### 3.2. Analysis of Thermal Properties

#### 3.2.1. Thermal Conductivity Properties of the Typical Materials in Residues

**Figure 5:**
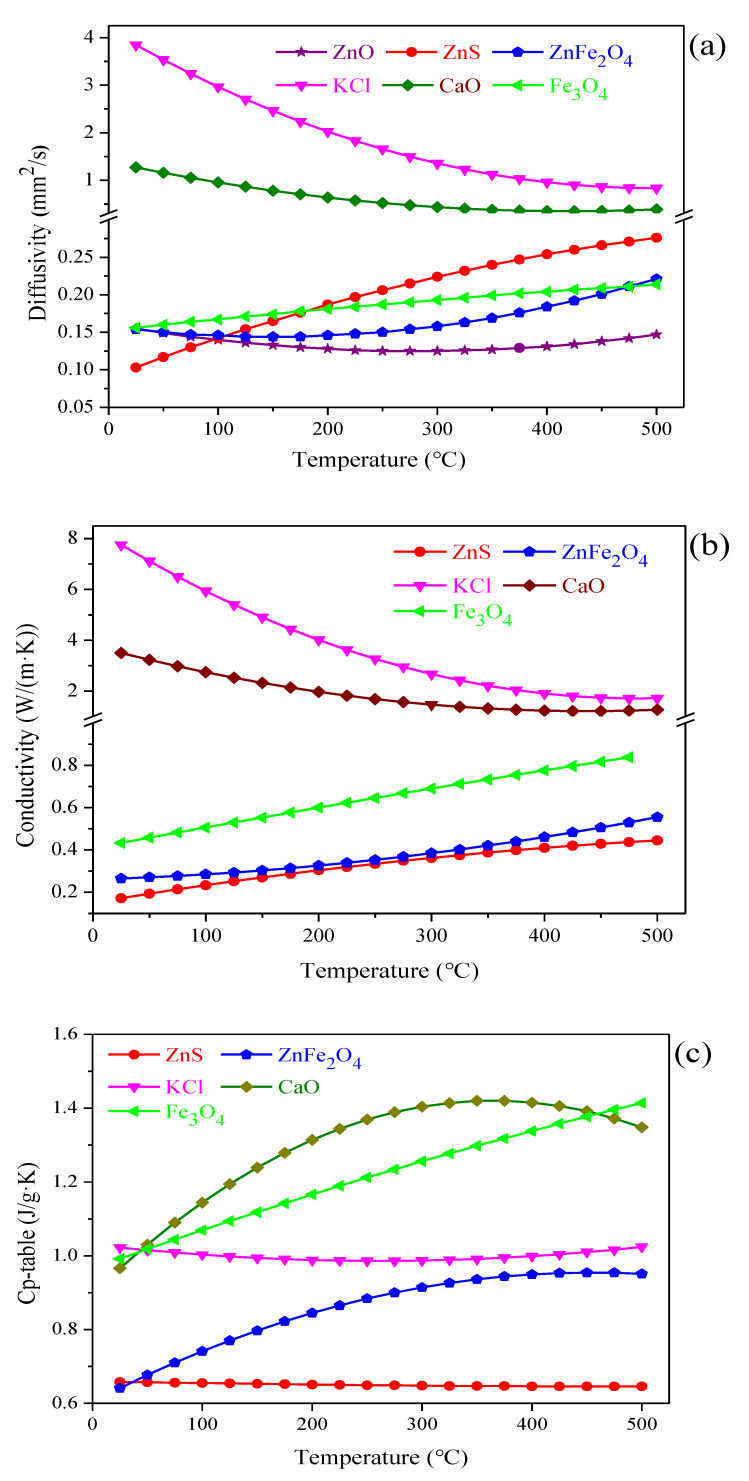
Effects of temperature on the thermal conductivity properties of the typical materials in the residues, (**a**) thermal diffusion coefficient; (**b**) thermal conductivity; (**c**) specific heat.

The thermal conductivity property is closely related to the composition, structure and temperature of the material. Based on the composition analysis results of zinc-containing residues (Table 1), the thermal conductivity properties of the typical materials in residues were measured in the temperature range of 25–500 °C, including ZnO, ZnS, ZnFe_2_O_4_, Fe_3_O_4_, KCl, and CaO. The parameters related to the apparent density of the typical materials are presented in Table 3. Besides, the change of the thermal diffusion coefficient, thermal conductivity, and specific heat of these typical materials in zinc-containing residues at different temperatures were investigated. The respective result is plotted in Figure 5a–c, respectively.

Regarding the thermal diffusion coefficient and thermal conductivity of those typical materials, it can be observed from Figure 5a,b that the two parameters of ZnS, ZnFe_2_O_4_, and Fe_3_O_4_ were lower than those of KCl and CaO, with the value below 0.5 and 1.0 respectively in the temperature range of 25–500 °C. This finding denoted that those materials including ZnS, ZnFe_2_O_4_ and Fe_3_O_4_ were of this type material whose thermal diffusion coefficient and thermal conductivity were relatively poor and showed a slight increase trend with a temperature increasing. There was absent of significant influence for temperature on the thermal conductivity properties. The changing trend of the thermal diffusion coefficient of ZnO was similar to that. However, the temperature of ZnO can increase faster with the increase of the interface temperature under high-temperature conditions. Meanwhile, it can be found that the temperature guiding efficiency of ZnFe_2_O_4_ was obviously lower, similar to that of heat preservation materials. Moreover, the thermal diffusion coefficient and thermal conductivity of KCl and CaO were relatively high at low temperatures, decreased sharply with a temperature rising, and became stable at a temperature higher than 400 °C. Furthermore, for the specific heat of the typical materials, it was observed from Figure 5c that in the measurement temperature range, the specific heat of CaO was the highest, followed by Fe_3_O_4_, KCl, ZnFe_2_O_4_, and ZnS. The changes of the specific heat of materials with the temperature increasing varied. Specifically, the specific heat of CaO first increased and then decreased with a temperature rising, and reached the maximum value at about 350 °C; the specific heat of Fe_3_O_4_ increased linearly with the increase of temperature; the specific heat of KCl first decreased and then increased with a temperature rising. However, the changing trend is not obvious; the specific heat of ZnFe_2_O_4_ first increased and then tended to be flat with the increase of temperature. The specific heat of ZnS did not change with the temperature changing, and was relatively low, indicating that the heat absorption capacity of ZnS is unaffected by temperature. In summary, CaO phase with excellent thermal conductivity properties can remedy the insufficient heating efficient caused by ZnS, ZnFe_2_O_4_, and Fe_3_O_4_ with poor thermal conductivity properties, further to improve the heating efficient during heating process.

#### 3.2.2. Influence of CaO Addition on the Thermal Conductivity Properties of Residues

**Figure 6:**
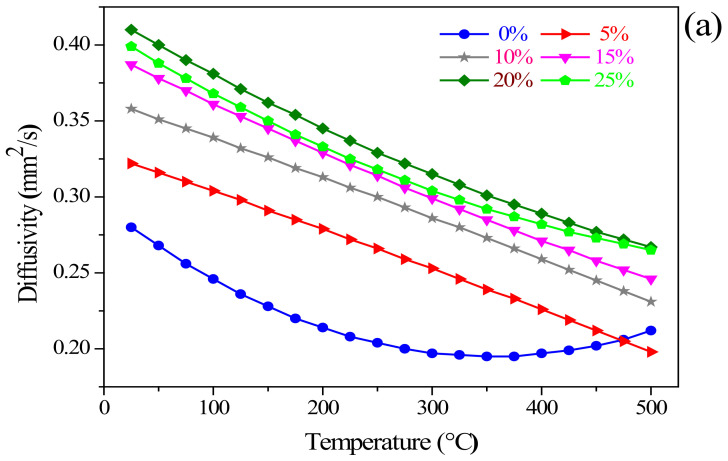

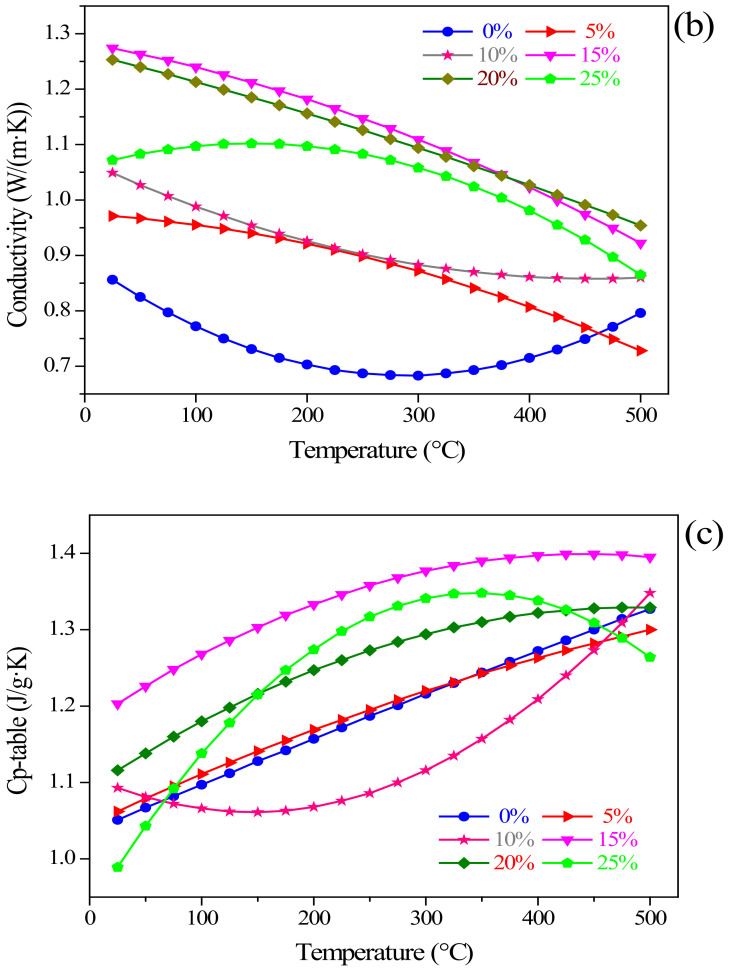
Thermal diffusion coefficient (**a**), thermal conductivity (**b**), and specific heat (**c**) of the residues with different CaO addition amounts.

Based on the thermodynamic characteristics analysis of calcification pretreatment for zinc-containing residues, the thermal conductivity properties of the residues added with 0%, 5%, 10%, 15%, 20% and 25% CaO were measured in the temperature range of 25–500 °C. The parameters related to the apparent density of the residues with different CaO addition amounts are displayed in Table 4. Besides, the change of the thermal diffusion coefficient, thermal conductivity, and specific heat of these typical materials in zinc-containing residues at different temperatures were investigated. The respective result is plotted in Figure 6a–c, respectively.

Regarding the thermal diffusion coefficient and thermal conductivity, it can be found from Figure 6a,b that the two parameters of the zinc-containing residues without CaO additives added first decreased and then increased with a temperature rising. However, after adding different amounts of CaO, the overall changing trend of the two parameters with different temperatures can be viewed as a decreasing trend with the temperature increasing. As illustrated in Figure 6b, the thermal conductivities of the residues added with 10% and 25% CaO were significantly affected by temperature. The thermal conductivity of the residues added with 10% CaO decreased rapidly with the temperature rising; however, the residues added with 25% CaO was just the opposite, showing a slower decreasing trend, which indicated that the residues added with 25% CaO endowed excellent thermal conductivity properties at a relatively low temperature. Under certain temperature conditions, the thermal diffusivity coefficient and thermal conductivity of zinc-containing residues increased first and then decreased with the increase of CaO addition. Besides, for the specific heat of the residues with different CaO additions, it was observed from Figure 6c that within the measurement temperature range, there was absent of obvious law with respect to temperature. Specifically, the specific heat of the residues added with 10% CaO first decreased and then increased with a temperature increasing; the specific heat of the residues added with 25% CaO increased first and then decreased with the temperature rising, and reached the maximum value of 1.348 J/g·K at 350 °C; additionally, the specific heat properties of the residues added with another CaO content improved with a temperature increasing and stabilized at temperatures exceeding 400 °C.

The thermal conductivity properties of zinc-containing residues were significantly affected by CaO addition amount and changes in temperature. Therefore, considering the influence of the two influencing factors on the phase transformation in the zinc-containing residues is of great significance for Zn recovery. From the above analysis, it could be speculated that the favorable CaO addition amount and temperature to be considered is near 25% and 350 °C, respectively.

### 3.3. Analysis of Microwave Heating Characteristics

**Figure 7:**
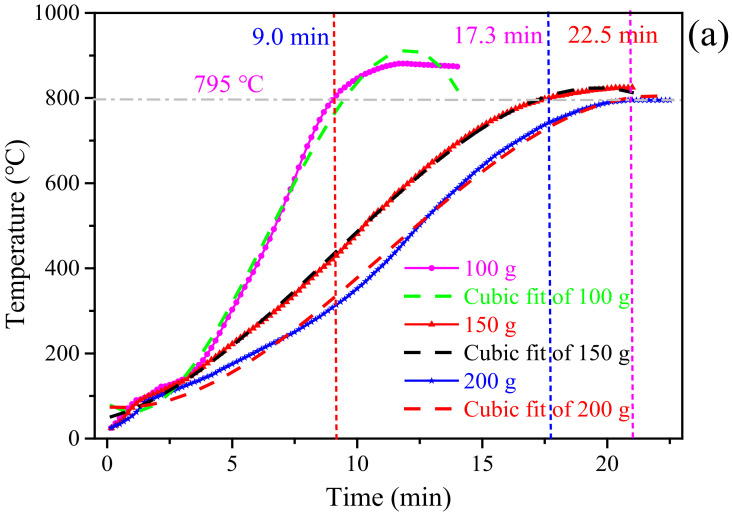

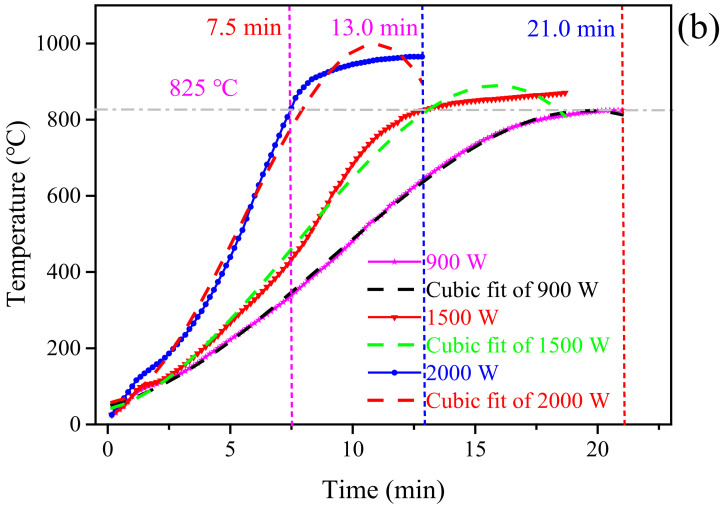
Microwave heating curves of the residues under (**a**) different sample qualities and (**b**) different microwave powers.

Knowing the microwave heating characteristics of the residues contributes to analyzing the dielectric properties of the material [41]. The microwave heating characteristics of the residues was measured at 2450 MHz with different sample qualities and different microwave powers. The results were illustrated in Figure 7.

The heating behavior of zinc-containing residues in the microwave field is closely related to the amount of material [42]. Under a microwave output power of 900 W, the influence of the residues amount on the heating behavior was depicted in Figure 7a. The empirical relationships between the temperature (*T*_m_) and time for the residues with a tested mass of 100 g, 150 g and 200 g were shown in Equations (1)–(3), respectively:

*T*_m_ = 83.8261 − 43.9448*t* + 24.4900*t*^2^ − 1.2568*t*^3^ (R^2^ = 0.9942) (1)

*T*_m_ = 48.6557 + 15.7905*t* + 4.4438*t*^2^ − 0.1649*t*^3^ (R^2^ = 0.9993) (2)

*T*_m_ = 74.6008 − 5.3662*t* + 5.0844*t*^2^ − 0.1515*t*^3^ (R^2^ = 0.9963) (3)

As presented in Figure 7a, the apparent average heating rate of the residues with a tested mass of 100, 150, and 200 g was 84.60 °C/min, 46.41 °C/min, and 39.45 °C/min, respectively. As the amount of material increases, the longer it takes to reach the same temperature. For the same target temperature of 795 °C, it took 9 min, 17.3 min, and 22.5 min for the residues with a tested mass of 100, 150, and 200 g, respectively. The thicker the material, the greater the resistance of internal microwave penetration is. During the interaction between microwaves and materials, a larger amount of material corresponds to a larger reflection peak, which is manifested by the increase in the reflectivity of the material to microwaves. According to the theory of microwave transmission line [43,44,45], the larger the amount of material, the thicker the heated sample is, the greater the reflection loss of microwave will be. The lower the utilization rate of microwave energy by the material will be.

Figure 7b illustrated the heating curves of the residues with a mass of 150 g under different microwave powers. The empirical relationship between the temperature (*T*_m_) and time for the residues under microwave powers of 900, 1500 and 2000 W was presented in Equations (4)–(6), respectively:

*T*_m_ = 48.6557 + 15.7905*t* + 4.4438*t*^2^ − 0.1649*t*^3^ (R^2^ = 0.9993) (4)

*T*_m_ = 40.4751 + 14.8002*t* + 8.2434*t*^2^ − 0.3659*t*^3^ (R^2^ = 0.9935) (5)

*T*_m_ = 57.4756 + 0.4315*t* + 23.6205*t*^2^ − 1.4456*t*^3^ (R^2^ = 0.9920) (6)

As presented in Figure 7b, the apparent average heating rate of the residues under microwave powers of 900, 1500, and 2000 W was 46.41, 67.00, and 110.63 °C/min, respectively. For the same target temperature of 825 °C, it took 7.5, 13.0, and 21.0 min for the residues under microwave powers of 900, 1500, and 2000 W, respectively. Meanwhile, the heating rate of the residues improved with the increase of microwave power. The temperature reached a higher value in the same time period, which denoted that the greater the microwave power, the faster the material heating rate will be. Under certain conditions, increasing the microwave output power means the enhancement in the electric field intensity [46,47,48]. As the electric field intensity (E) increases, the microwave can penetrate into the roasting material better and evenly, increasing the microwave penetration of zinc-containing residues and promoting the absorption of microwave power, further to make the material heat up quickly [49,50]. Therefore, appropriately increasing the microwave power can shorten the heating time and increase the apparent average heating rate of zinc-containing residues.

### 3.4. Analysis of Dielectric Properties

**Figure 8:**
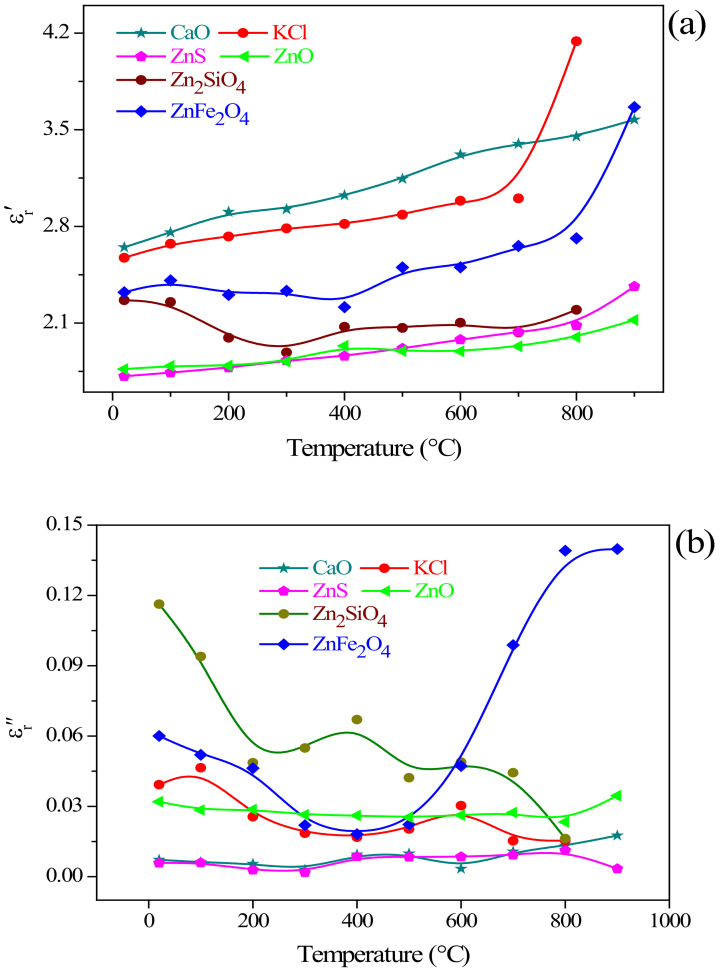

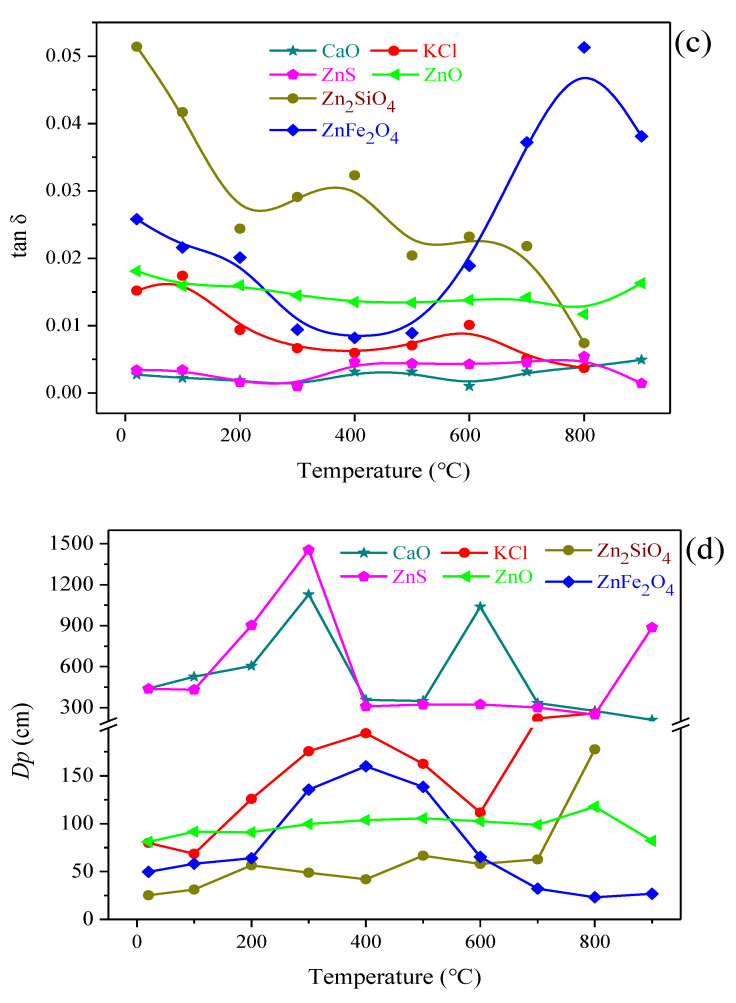
Dielectric properties of the typical materials in the residues, (**a**) dielectric constant (ε_r_′); (**b**) dielectric loss factor (ε_r_″); (**c**) loss tangent coefficient (tan δ); (**d**) microwave penetration depth (D_p_).

**Figure 9:**
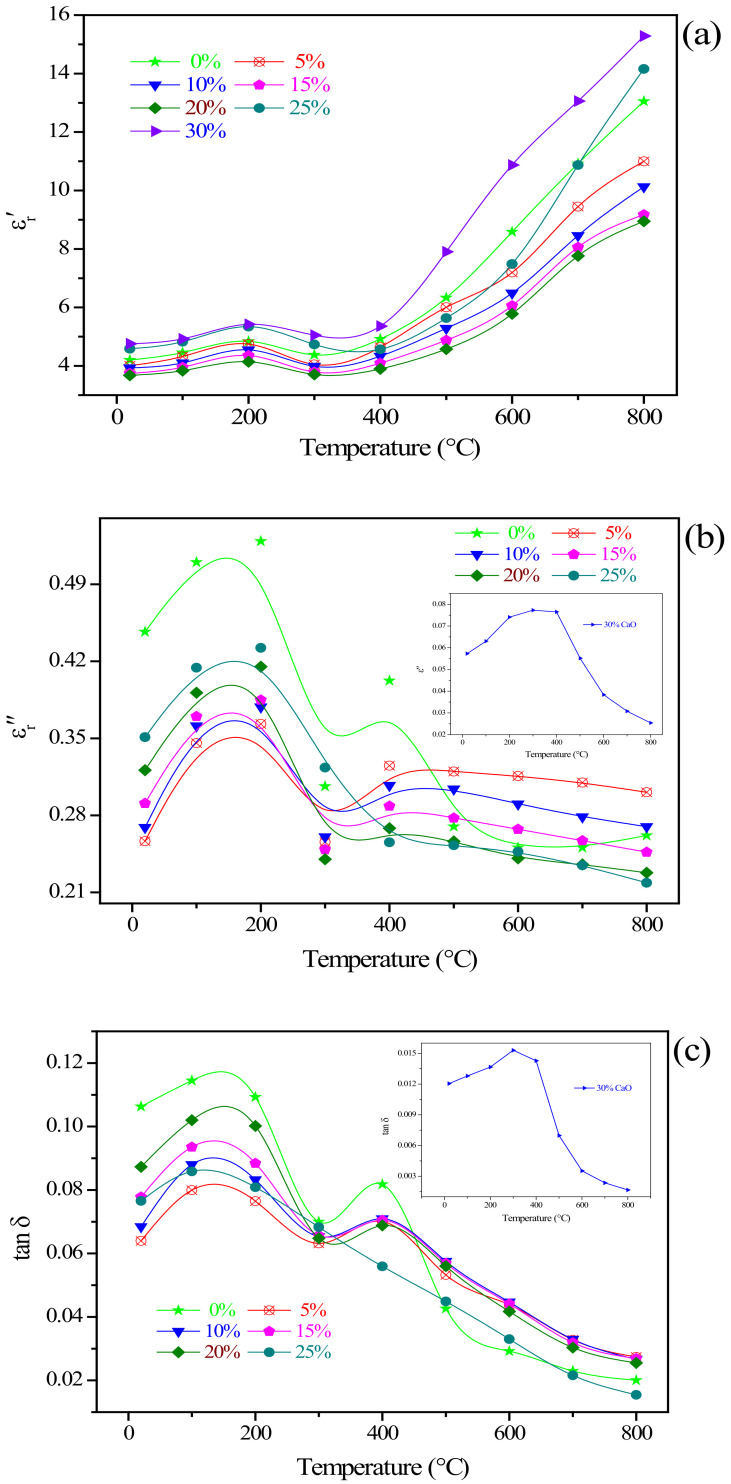

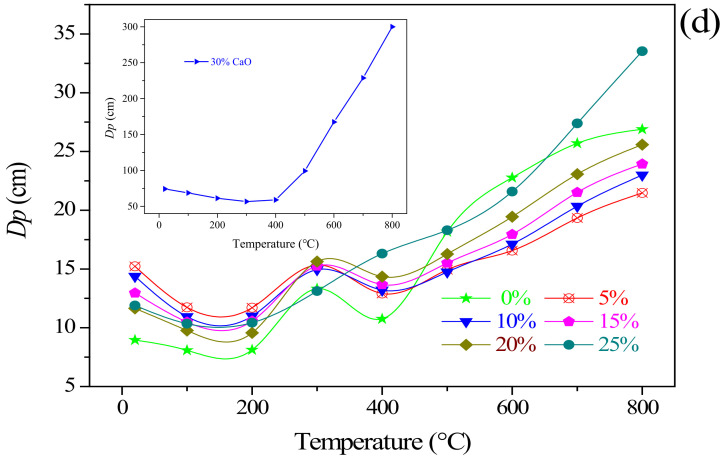
Dielectric properties of the residues with different CaO addition amounts, (**a**) dielectric constant (ε_r_′); (**b**) dielectric loss factor (ε_r_″); (**c**) loss tangent coefficient (tan δ); (**d**) microwave penetration depth (D_p_).

The dielectric properties of the typical phases in the residues and the residues added with different CaO additions were measured at 2450 MHz, including the dielectric constant (ε_r_′), dielectric loss factor (ε_r_″), loss tangent coefficient (tan δ), and penetration depth (D_p_). The respective results were plotted in Figure 8 and Figure 9, respectively.

#### 3.4.1. Dielectric Properties of the Typical Materials in Residues

Combining with the raw material characterization analysis of the residues and the thermodynamic analysis of CaO addition for the refractory ore phase transformation, the composition of zinc-containing residues is complex. Therefore, before studying the influence of CaO addition on the dielectric properties of the residues, the change of dielectric properties of the single-phase in residues should be investigated, including ZnO, ZnFe_2_O_4_, Zn_2_SiO_4_, ZnS, KCl, and CaO. The measured results are shown in Figure 8.

As illustrated in Figure 8, the dielectric constant and dielectric loss of ZnO and ZnS were equivalent, and were significantly lower than the same parameter values of ZnFe_2_O_4_, Zn_2_SiO_4_, KCl, and CaO. It can be observed from Figure 8a that the dielectric constant of those single substances in zinc-containing residues improved significantly with the increase of temperature, that is, the ability to absorb and store microwaves enhances with the increase of temperature. The effect of temperature on the dielectric constant is essentially the effect of temperature on the dielectric relaxation process. The dielectric relaxation time will decrease with the increase of temperature, resulting in an increase in the dielectric constant. Among the several types of single substance, the dielectric constant of CaO is significantly higher than that of other substances, indicating that the addition of CaO can enhance the microwave absorption and storage capacity of zinc-containing residues, and it gradually strengthens with the increase of temperature.

For the dielectric loss factor (ε_r_″), loss tangent coefficient (tan δ), it can be found from Figure 8b,c that the dielectric loss and loss tangent values of ZnO, ZnS, and CaO remained unchanged, while the change patterns of ZnFe_2_O_4_, Zn_2_SiO_4_, and KCl were quite different. The dielectric loss and loss tangent value of Zn_2_SiO_4_ greatly decreased with a temperature increasing. Meanwhile, in the range of 25–400 °C, the dielectric loss and loss tangent value of ZnFe_2_O_4_ also decreased with increasing temperature; at temperature higher than 400 °C, the two parameters values of ZnFe_2_O_4_ continued to increase with a temperature increasing. In general, the dielectric properties of ZnFe_2_O_4_ were significantly affected by the temperature. The decrease in dielectric loss and loss tangent value with the increase of temperature indicates that the material is easy to obtain better uniformity of microwave heating during the microwave heating process.

Figure 8d depicted the characteristics of the microwave penetration depth of ZnO, ZnFe_2_O_4_, Zn_2_SiO_4_, ZnS, and CaO as a function of temperature. The microwave penetration depth of ZnO, ZnFe_2_O_4_, Zn_2_SiO_4_, and KCl changed slightly with the increase of temperature. Conversely, the penetration depth of ZnS and CaO varied significantly with the increase of temperature: the penetration depth of ZnS and CaO had obvious peaks at 300 °C, reaching 1454.62 and 1128.5 cm, respectively; at temperature rising to 400 °C, the microwave penetration depth of ZnS and CaO decreased significantly; and there arose a relatively stable equilibrium stage at 400–500 °C, with the microwave penetration depth of 309.24 and 256.88 cm, respectively; with the temperature continuously rising, the microwave penetration depth of ZnS and CaO both increased and decreased. Generally, in the considered temperature range (25–900 °C), the microwave penetration depth of the typical materials studied is larger. The material with a larger microwave penetration depth is a necessary condition to obtain the uniformity of microwave heating.

#### 3.4.2. Influence of CaO Addition on the Dielectric Properties of Residues

The dielectric properties of zinc-containing residues added with different CaO were investigated. The results are illustrated in Figure 9. For the dielectric constant, it was concluded from Figure 9a that in the range of 25–400 °C, adding 5–20% CaO presented little effect on the dielectric constant of zinc-containing residues; however, as the CaO addition amount increased to 25%, the dielectric constant increased significantly. Besides, at a temperature higher than 400 °C, the dielectric constant values of the zinc-containing residues raw materials and the mixed residues added CaO improved significantly with the temperature increasing. This change law was consistent with the heating characteristics of the residues: the heating rate of the residues below 400 °C was relatively slow, that is, the ability to absorb and store microwaves is relatively weak; however, the temperature rise rate was significantly increased at a temperature exceeding 400 °C.

Figure 9b,c described the change of dielectric loss and loss tangent of zinc-containing residues added with 0–30% CaO with different temperatures, respectively. As presented in Figure 9b, the dielectric loss of zinc-containing residues added with 0–20% CaO can be divided into three stages by temperature: (1) The first stage at 25–300 °C, there is a trend of increasing first and then decreasing. This stage can be attributed to the homogenized heat transfer process of the zinc-containing residues that absorbs microwaves and converts microwaves into heat energy. (2) The second stage at 300–500 °C, there is also a tendency to increase first and then decrease. This change indicated the phase structure may change during the microwave heating process. Based on the above thermodynamic characteristics analysis, at a temperature higher than 400 °C, ZnFe_2_O_4_ and Zn_2_SiO_4_ react with CaO and transform into a large amount of ZnO. As shown in Figure 8a, ZnO is a weakly microwave-absorbing substance, and the dielectric constant value of ZnO is significantly lower than that of ZnFe_2_O_4_ and Zn_2_SiO_4_. The large amount of new generated ZnO phase reduced the ability of the whole residues to absorb microwaves and convert them into heat. Therefore, the dielectric loss of the material also increased with the temperature as the reaction proceeds. (3) The third stage at a temperature higher than 500 °C, the dielectric loss of the residues tended to be stable, indicating that there was absent of obvious thermal conversion of microwave energy at this stage, that is, the phase transformation of refractory mineral phase arose at the temperature range of 300–500 °C. It is worth noting that, the change in the dielectric loss of zinc-containing residues added with 25% CaO included only two stages, and it tended to balance around 400 °C. The decrease in the equilibrium temperature may be attributed to the increase in CaO content makes the phase inversion reaction is more rapid at the same temperature. Besides, the dielectric loss of the residues added with 30% CaO was significantly lower than other residues with other CaO additions. The temperature change showed no significant effect on the dielectric loss.

For the loss tangent coefficient, it was found from Figure 9c that in the range of 25–400 °C, the changing trend of the loss tangent coefficient of the residues with different CaO contents was similar to the dielectric loss changes with the temperature. At a temperature exceeding 400 °C, the loss tangent coefficient decreased sharply with the increase of temperature. This phenomenon is assigned to the dielectric loss remained unchanged within this temperature range, while the dielectric constant rose sharply; therefore, the ratio of the dielectric loss to the dielectric constant (i.e., the loss tangent coefficient) showed a decreasing trend with the temperature increasing. For the microwave penetration depth, it can be concluded from Figure 9d that in the range of 25–800 °C, there was a significant difference in the microwave penetration depth of the residues with 0–25% CaO and 30% CaO. The penetration depth of the residues added with 0–25% CaO was between 7–35 cm and increased with the temperature rising. However, for the residues added with 0–20% CaO, there arose two troughs in the range of 25 °C–500 °C. This finding denoted that adding 5–20% CaO and no adding CaO had the same effect on the microwave penetration depth; meanwhile, the microwave penetration ability was strengthened with the addition of CaO content; moreover, under the condition that the thickness of the baked material without exceeding the microwave penetration depth, the high microwave penetration depth is the prerequisite to ensure the uniformity of microwave heating. Furthermore, the microwave penetration depth of the residues added with 25% CaO did not decrease with the increase of temperature in the range of 300–500 °C, conversely promoted the microwave penetration depth increased, which indicated that adding 25% CaO within the range of 300 °C–500 °C presented a positive effect on improving the uniformity of microwave heating. However, it was found from Figure 9d that in the range of 25–400 °C, the microwave penetration depth of the residues added with 30% CaO did not increase with the increase of temperature, the microwave penetration depth was maintained in the range of 50–75 cm; at a temperature higher than 400 °C, the increase in temperature showed a significant effect on the microwave penetration depth. The penetration depth of microwaves can reach 300 cm at 800 °C. Combining with the analysis of Figure 9b,c, it can be summarized that the dielectric loss and loss tangent coefficient of the residues added with 30% CaO significantly reduced, indicating the utilization rate of microwave energy on minerals was significantly reduced. The time required to reach the required temperature under the same conditions will be correspondingly extended. Therefore, it was summed that the amount of CaO additive should not be higher than 30% for the effective use of microwave energy for microwave mineral activation pretreatment. The favorable CaO addition amount was determined as 25%.

## 4. Conclusions

In this study, a activate pretreatment approach was proposed with calcium oxide (CaO) as activator and introducing microwave heating instead of conventional heating, expecting to realize the conversion of refractory ore phase (including ZnS, ZnFe_2_O_4_, and Zn_2_SiO_4_) into pre-treated ore phase. Meanwhile, the calcification thermodynamics characteristics, thermal conductivity properties and dielectric properties of calcium-activated zinc-containing residues were determined to clarify the feasibility of the proposed approach. The main conclusions were drawn as following:

(1) Compared with the carbothermal reduction reaction (with the addition of C), Zn_2_SiO_4_ and ZnFe_2_O_4_ phases can also undergo mineral phase conversion under the roasting conditions with the addition of CaO; meanwhile, ZnS phase can also undergo oxidation reactions in the air atmosphere during roasting.
(2) The thermal conductivity properties of ZnS and ZnFe_2_O_4_ were relatively poor than that of CaO phase, with the value below 0.5 and 1.0 respectively in the temperature range of 25–500 °C, thereby adding CaO activator can remedy and enhance the heating efficient of residues during heating process. Besides, the thermal conductivity properties of the residues added with 25% CaO were significantly superior than the residues added with other CaO contents, with the maximum specific heat value of 1.348 J/g·K at 350 °C.
(3) The dielectric constant properties of CaO activator were higher than that of other substances; hence, the addition of CaO can enhance the microwave-absorbing ability of zinc-containing residues. Meanwhile, the dielectric loss and loss tangent values decreased with a temperature increasing and the residues had large microwave penetration depth, which guaranteed the better uniformity of microwave heating. Furthermore, adding 25% CaO promoted the microwave penetration depth of the residues increased in the range of 300–500 °C.

## Figures and Tables

**Table 1:** The chemical compositions of the residues.

Compositions	Zn	Fe	C	Si	CaO	Al_2_O_3_
Mass (w%)	24.74	21.66	9.14	2.66	4.1	2.22
**Compositions**	**Cl**	**S**	**Mg**	**Bi**	**Pb**	**In (g/t)**
Mass (w%)	2.94	1.39	1.14	0.97	1.13	354

**Table 2:** The main reactions probably appeared in the calcium-activated residues.

NO.	Reactions	NO.	Reactions
1	ZnO(s) + Fe_2_O_3_(s) = ZnFe_2_O_4_(s)	2	ZnO(s) + C(s) = Zn(g) + CO(g)
3	ZnO(s) + CO(g) = Zn(g) + CO_2_(g)	4	Zn_2_SiO_4_(s) + CaO(s) = 2ZnO(s) + CaSiO_3_(s)
5	Zn_2_SiO_4_(s) + 2C(s) = 2Zn(g) + SiO_2_(s) + 2CO(g)	6	ZnS(s) + CaO(s) = ZnO(s) + CaS(s)
7	ZnS(s) + CaO(s) + C(s) = Zn(g) + CaS(s) + CO(g)	8	2ZnS(s) + 3O_2_(g) = 2ZnO(s) + 2SO_2_(g)
9	3ZnFe_2_O_4_(s) + C(s) = 3ZnO(s) + 2Fe_3_O_4_(s) + CO(g)	10	ZnFe_2_O_4_(s) + 2C(s) = Zn(g) + 2FeO(s) + 2CO(g)
11	3ZnFe_2_O_4_(s) + 4C(s) = 3Zn(g) + 2Fe_3_O_4_(s) + 4CO(g)	12	ZnFe_2_O_4_(s) + 4C(s) = Zn(g) + 2Fe(s) + 4CO(g)
13	ZnFe_2_O_4_(s) + 2CaO(s) = ZnO(s)+ Ca_2_Fe_2_O_5_ (s)	14	CaO(s) + CO_2_(g) = CaCO_3_(s)

**Table 3:** Parameters related to the apparent density of the typical materials.

Materials	Mass (g)	Thickness (mm)	Density (g/mm^3^)
ZnO	0.1023	2.00	0.54
ZnS	0.6050	2.42	2.50
ZnFe_2_O_4_	0.4998	1.89	2.65
Fe_3_O_4_	0.5936	2.09	2.84
KCl	0.4359	2.18	2.00
CaO	0.4597	1.91	2.41

**Table 4:** Parameters related to the apparent density of the residues with different CaO addition amounts.

CaO Addition Amounts	Mass(g)	Thickness(mm)	Density(g/mm^3^)
0%	0.5129	1.80	2.85
5%	0.5882	2.08	2.83
10%	0.6679	2.42	2.76
15%	0.5930	2.20	2.70
20%	0.6862	2.55	2.69
25%	0.6095	2.35	2.59

## Data Availability

The data presented in this study are available on request from the corresponding author. The data are not publicly available due to technical or time limitations.

## Supplementary Materials

The following supporting information can be downloaded at: https://www.mdpi.com/article/10.3390/ma15030714/s1, Section S1: Measurement Principle for Thermal Conductivity; Section S2: Measurement Principle for Dielectric Property.
