# Supplementary material for: Investigations on the Thermodynamics Characteristics, Thermal and Dielectric Properties of Calcium-Activated Zinc-Containing Metallurgical Residues
Source: Materials (Basel). 2022 Jan 18;15(3):714. doi: 10.3390/ma15030714 (PMC8836694; doi:10.3390/ma15030714)
Supplement: Supplementary file 1 [file materials-15-00714-s001.zip › Supplementry Materials.pdf]

Investigations on the Thermodynamics Characteristics, Thermal and Dielectric Properties of Calcium-Activated Zinc-Containing Metallurgical Residues

Aiyuan Ma [1,2], Xuemei Zheng [1,2], Lei Gao [3], Kangqiang Li [3], Mamdouh Omran [4,5*] and Guo Chen [3*]

[1] School of Chemistry and Materials Engineering, Liupanshui Normal University, Liupanshui 553004, China
[2] Guizhou Provincial Key Laboratory of Coal Clean Utilisation, Liupanshui, 553004, China
[3] Key Laboratory of Green-Chemistry Materials in University of Yunnan Province, Kunming Key Laboratory of Energy Materials Chemistry, Yunnan Minzu University, Kunming 650500, China
[4] Process Metallurgy Research Group, Faculty of Technology, University of Oulu, Finland
[5] Central Metallurgical Research and Development Institute (CMRDI), Cairo 11421, Egypt
* Correspondence: Corresponding author: mamdouh.omran@oulu.fi
* Correspondence: Corresponding author: guochen@kust.edu.cn

S1. Measurement Principle for Thermal Conductivity

The thermal conductivity performance of minerals is an important factor affecting the heat treatment process, and it is related to those factors such as thermal conductivity (λ), thermal diffusion coefficient (a), and specific heat (c) of the material. In this work, the thermal conductivity parameters of the MSD sample were measured using the laser flash analyzer (LFA 467, NETZSCH, Germany) through the laser flash method. The laser flash method (GB/T 22588-2008) is a common method used to measure the thermal conductivity of materials, which belongs to the transient method. The measurement principle is that at a specific set temperature (T), a light pulse is instantly emitted by the laser source and uniformly illuminates the lower surface of the sample, so that the surface layer absorbs light energy and the temperature rises instantaneously, and acts as the hot end to spread the energy to the cold end (upper surface) in one-dimensional heat conduction mode. Followed by the infrared detector is used to continuously measure the corresponding temperature rising process at the center of the upper surface to obtain the temperature rising curves versus time. If the light pulse width is close to infinitely small or approximately negligible relative to the half heating time of the sample, the heat conduction process inside the sample is an ideal one-dimensional heat transfer from the lower surface to the upper surface, with no lateral heat flow; meanwhile, the sample absorbs the radiation under the ideal condition that the temperature rises uniformly and there is no heat loss, the thermal diffusion coefficient (α) of the sample at temperature T can be expressed as follows:

$$\alpha=0.1388*d^2/t_{50} \tag{S1}$$

where d is the thickness of the sample; t_{50} is the half-heating time, that is, the time required for the upper surface temperature of the sample to rise to half of the maximum value.

The conversion relationships among the thermal conductivity (λ) and thermal diffusivity (α), specific heat (C), and apparent density (ϱ) are defined as follows:

$$\lambda(T)=\alpha(T)*Cp(T)*\rho(T) \quad (S2)$$

Knowing the thermal diffusivity, specific heat and density of materials at a certain temperature (T), the thermal conductivity (λ) at the temperature (T) can be calculated. The apparent density (ρ) is used as the volume density, which is the ratio of mass to apparent volume. It (ρ) is generally tested at room temperature, and its change with temperature can be corrected using the thermal expansion coefficients of the material; meanwhile, it can be approximately regarded as unchanged when the measured temperature is not too high and the density change is not too large. If the sample has a regular shape and a smooth surface, the specific heat (Cp) can be measured together with the thermal diffusivity by the LFA 467 device.

S2. Measurement Principle for Dielectric Property

The interaction between microwave and the heated material is expressed by complex permittivity. Usually, the real part (ε_r') of the complex permittivity indicates the material's polarization ability in the microwave field, also namely dielectric constant; the imaginary part (ε_r'') of the complex permittivity denotes the material's thermal loss ability in the microwave field, also namely dielectric loss factor; and the loss tangent coefficient (tan δ) is characterized by the ratio of the imaginary part (ε_r') to the real part (ε_r'') of the complex permittivity, which describes the ratio of thermal loss power to reactive power caused by polarization. The involved equations were displayed as follows:

$$\varepsilon(T)=\varepsilon_0[\varepsilon_r'(T)\text{-}i\varepsilon_r''(T)] \quad (S3)$$

$$tan\delta = \varepsilon_r'' / \varepsilon_r' \quad (S4)$$

where ε is the complex permittivity, F/M; ε_0 is the permittivity under vacuum conditions (there is no loss) , F/M; ε_r' is the relative permittivity, F/M; i is the imaginary unit $(-1)^{1/2}$; ε_r'' is the dielectric loss, F/M. Both the dielectric constant and the dielectric loss are functions of temperature, and the loss tangent is the ratio of the two, so the loss tangent is also a function of temperature.

The resonant cavity perturbation method is widely used in the measurement of the complex permittivity of solid and liquid materials. The basic concept is that the shift of resonant frequency of resonant cavity and decrease of Q value caused by loading a small sample into the resonant cavity, wherein the real part of the complex permittivity can be obtained from the change of the resonant frequency, and the imaginary part can be obtained from the change of the resonant cavity Q value. In practical applications, the resonant cavity is mostly rectangular or cylindrical. The dielectric properties measurement involved in this work adopted the cylindrical resonant cavity test method. For a cylindrical cavity, the electric and magnetic fields of the TM_{0n0} mode are respectively concentrated near the central axis of the cylindrical cavity and near the inner wall of the cylindrical cavity. Therefore, the TM_{0n0} mode is suitable for testing the complex permittivity in the cylindrical cavity.
